# Nurse-Led Sepsis Scoring System for Adult Febrile Neutropenia: Protocol for a Two-Phase Clinical Prediction Model Study With Remote Patient Monitoring

**DOI:** 10.2196/107468

**Published:** 2026-11-05

**Authors:** N Aswathy Asokan, K L Ajee, Sachin Jadhav, Nancy Dias, B Premjith, K T Moly

**Affiliations:** 1 Department of Medical Surgical Nursing, Amrita College of Nursing, Amrita Vishwa Vidyapeetham, Kochi, Kerala, India; 2 Department of Medical Surgical Nursing, Amrita College of Nursing, Amrita Vishwa Vidyapeetham, Health Sciences Campus, Edappally, Kochi, Kerala, 682041, India, 91 8335053920; 3 Department of Hematology and the BMT, Trustwell Hospitals, Bengaluru, Karnataka, India; 4 Department of Nurse Science, East Carolina University, Greenville, NC, United States; 5 Department of Artificial Intelligence, Amrita School of Artificial Intelligence, Amrita Vishwa Vidyapeetham, Coimbatore, Tamil Nadu, India; 6 Department of Medical Surgical Nursing, Amrita College of Nursing, Amrita Vishwa Vidyapeetham, Health Sciences Campus, Kochi, Kerala, India

**Keywords:** febrile neutropenia, sepsis, hematologic malignancies, clinical prediction model, nurse-led scoring system, remote patient monitoring, artificial intelligence, supportive oncology nursing, temporal validation, digital health

## Abstract

**Background:**

Adults with hematological malignancies who develop febrile neutropenia (FN) face elevated risks of sepsis, intensive care admission, and treatment interruptions. Early sepsis detection is challenging, as classical clinical signs are often attenuated or delayed in this population. Current risk stratification tools demonstrate limitations when applied to adults with FN. A tailored, nurse-led scoring system integrating clinical, laboratory, and physiological predictors may enhance early sepsis identification.

**Objective:**

This 2-phase study aims to develop and internally validate a nurse-led sepsis risk score for adults with FN in phase I and to temporally validate its performance in phase II. The new score will be compared to the MASCC risk index, CISNE, NEWS2_ and qSOFA at FN onset . The secondary objectives include evaluating associations between risk groups and remote patient monitoring alerts within 24 hours of FN onset, interrater agreement, and bedside feasibility.

**Methods:**

The study is being conducted in the hematology and bone marrow transplantation department of a tertiary care hospital in Bengaluru, India. Phase I uses a nested matched case-control design within a retrospective cohort of approximately 140 patients with FN. We will analyze clinical, laboratory, and physiological predictors measured at FN onset using multivariable conditional logistic regression, with internal validation via bootstrapping. In Phase II, which utilizes a prospective temporal validation cohort, bedside nurses will calculate the proposed score at FN onset alongside the MASCC, CISNE, NEWS2, and qSOFA scores A substudy will include additional nurses scoring independently to assess interrater agreement and feasibility. Two blinded reviewers will adjudicate phase I outcomes within 14 days of FN onset, while assessors blinded to all scores will adjudicate phase II outcomes within the same time frame. The primary outcome is sepsis within 14 days of FN onset. Phase II evaluates discrimination, calibration, and comparative diagnostic performance.

**Results:**

We obtained ethics approval in April 2025, and the study was registered in June 2025 (CTRI/2025/06/088290). The initial phase I derivation was completed in November 2025, using sepsis within 14 days of FN onset as the primary outcome. A protocol amendment in September 2026 harmonized the outcome window to 14 days, prompting rederivation of the model and superseding the initial analysis. Phase II commenced in December 2025. As of September 2026, enrollment and data collection were ongoing, with study completion anticipated in December 2026. Main results are expected in 2027.

**Conclusions:**

This protocol evaluates an FN-specific, nurse-led sepsis score integrating clinical and laboratory predictors with contactless physiological measurements. We will examine remote patient monitoring alerts as secondary markers of clinical instability. Validation findings will establish the score’s predictive performance and inform future implementation studies.

## Introduction

Febrile neutropenia (FN), defined as a fever exceeding 38 °C with an absolute neutrophil count (ANC) below 500 cells per microliter, is one of the most serious oncologic emergencies encountered during cancer treatment [1-3]. It commonly occurs in patients receiving chemotherapy or undergoing hematopoietic stem cell transplantation. It affects an estimated 10% to 50% of individuals with solid tumors and up to 80% of those with hematological malignancies, with a similar incidence reported among patients with cancer in India [2]. FN promptly increases morbidity and mortality, is associated with approximately 2-fold higher rates of intensive care unit (ICU) admission, and can raise mortality to approximately 10% in high-risk groups [4]. Mortality is not uniform across contexts: rates are lower, approximately 1% to 5%, in low-risk solid tumor populations but rise to approximately 11% in hematological malignancies and increase further when patients are complicated by bloodstream infections, particularly Gram-negative bacteremia [5]. Across studies, older age, multiple comorbidities, intensive chemotherapy regimens, lowered performance status, and unfavorable baseline hematological profiles consistently emerge as predictors of poorer FN outcomes [6].

In FN, the underlying biology largely results from the treatment patients receive. Chemotherapy suppresses bone marrow activity, reducing neutrophil production and impairing neutrophil function [7,8]. At the same time, damage to the gastrointestinal and respiratory mucosa facilitates the entry of bacteria and other microbes into the bloodstream, increasing the risk of bloodstream infection [9,10]. In parallel, treatment-related immune disruption and altered cytokine responses weaken normal defense mechanisms and create conditions in which infections can develop and spread rapidly [11]. Unlike immunocompetent individuals, patients with FN often have muted inflammatory responses due to immunosuppressive therapies, which may mask classic clinical signs and delay infection diagnosis. Consequently, infections will progress to sepsis and septic shock, leading to multiorgan dysfunction, complicating early diagnosis and early intervention [12,13].

Sepsis frequently imposes a substantial clinical and economic burden on patients with cancer. Progression to septic shock needs advanced organ support, such as mechanical ventilation, vasopressors, and even renal replacement therapy, resulting in prolonged ICU stays and overall high treatment costs [14,15]. These complications are associated with higher death rates and can disrupt ongoing cancer treatment, resulting in delays or dose reductions in chemotherapy, leading to poor clinical outcomes. In this vulnerable group, recognizing sepsis at the earliest possible stage and initiating appropriate treatment without delay is essential both to improve survival and to keep cancer therapy as close to the planned course as clinically feasible [16-18].

Early detection of sepsis in FN is difficult because patients often present atypically. Fever may be the only early manifestation, while hallmark signs such as tachycardia, hypotension, tachypnea, and altered mental status can be absent or appear late. The clinical course is frequently rapid and unpredictable, leaving a narrow window for intervention [12,19]. Conventional monitoring strategies that rely on intermittent vital sign checks can miss subtle early physiological shifts, delaying sepsis recognition and reducing opportunities for timely escalation of care [5,20].

Several bedside scoring tools are available to help clinical teams judge risk and detect clinical deterioration in acute illness, including the Multinational Association for Supportive Care in Cancer (MASCC) score, the Clinical Index of Stable Febrile Neutropenia (CISNE), the National Early Warning Score 2 (NEWS-2), and the quick Sequential Organ Failure Assessment (qSOFA) [21-23]. However, when these tools are applied to patients with FN, important limitations emerge. MASCC and CISNE were developed to predict serious complications rather than to detect early sepsis, and their performance in hematological malignancies and other high-risk settings is frequently only moderately sensitive and shows imperfect discrimination [24]. NEWS-2 and qSOFA rely on overt physiological abnormalities and established organ dysfunction and have mainly been evaluated as predictors of sepsis-related mortality and ICU admission, rather than as tools for detecting the earliest stages of FN-related sepsis [23,25]. Importantly, none of these models contains FN-specific pathophysiological factors or the depth of immunosuppression, which likely contributes to poor predictive performance in this immunocompromised population.

Nurses hold a central role in recognizing sepsis early through continuous bedside presence and responsibility for patient monitoring, particularly in oncology and hematology settings. However, variations in clinical judgment, high patient loads, and workload pressures can delay recognition in practice. The lack of clear, nurse-focused decision support tools also contributes to inconsistencies in how FN-related sepsis risk is assessed, highlighting the need for a structured bedside scoring system designed specifically for patients with neutropenia [26,27].

Recent advances in AI and remote patient monitoring have created new options for continuous tracking of patients’ physiological status [28]. Contactless systems, including ballistocardiograph-based and video-derived vital sign frameworks, can monitor heart rate, respiratory rate, blood pressure, oxygen saturation, and temperature in real time, making it easier to recognize subtle changes that precede obvious clinical deterioration [29]. These technologies are especially useful in FN, where early warning signs may be muted or absent, and classical clinical indicators can remain silent. Despite this potential, AI-enabled remote monitoring has so far been incorporated only to a limited extent into routine FN workflows and sepsis prediction models for this group [30].

Overall, important gaps remain. No early-warning system is designed specifically for FN; widely used scores lack the sensitivity needed in this immunocompromised group, and newer options, such as AI-supported uninterrupted monitoring, are not yet routinely used to guide sepsis risk assessment. Developing an FN-specific, nurse-led scoring system that integrates clinical parameters with real-time physiological data may augment early sepsis detection, support more timely escalation, and ultimately reduce morbidity and mortality in this high-risk population. Framing the score as a complementary oncology care tool situates it within contemporary practice, intending to prevent severe infection-related complications, maintain chemotherapy continuity, limit avoidable ICU exposure, and improve quality of life for patients with hematological malignancies. In this study, nurse-led refers to both the nursing-driven design and development of the scoring system by a PhD nursing scholar and senior bone marrow transplantation (BMT)–hematology nurse and its planned application by frontline nursing staff at the bedside. Accordingly, phase II evaluates the score as applied by trained ward nurses rather than only by the research team and includes a nested assessment of interrater agreement and bedside feasibility (see “Two-Phase Study Framework” section). Subsequent implementation studies will evaluate downstream outcomes, including effects on workload, escalation behavior, and clinical outcomes.

This protocol has phase-specific primary and secondary objectives. In phase I, the primary objective is to identify predictive variables related to sepsis among adult patients with FN; the secondary objective is to develop and internally validate a nurse-led scoring system for early sepsis identification using these variables. In phase II, the primary objective is to compare the diagnostic performance of the nurse-led scoring system with established scoring tools (MASCC, CISNE, NEWS-2, and qSOFA) for early sepsis identification in adult patients with FN. Secondary phase II objectives are to assess the association between baseline risk groups derived from the nurse-led score and RPM alerts within 24 hours of FN onset and to evaluate interrater agreement and bedside feasibility when the score is applied independently by frontline ward nurses; we introduced this latter objective via protocol amendment in September 2026.

## Methods

### Study Design and Reporting Framework

This investigation is a multiphase translational study to develop and temporally validate a nurse-led clinical scoring system for the early identification of sepsis in adult patients with FN. The scoring system integrates routinely available clinical and laboratory data with physiological measurements from an AI-enabled, contactless remote patient monitoring (RPM) system. The RPM’s role differs by phase: in phase I, it functions as an accurate, nurse-independent instrument for capturing single point-in-time vital signs at the onset of FN, which then enter the model as candidate predictors. In phase II, continuous tracking is also used to detect clinically significant RPM alerts within the first 24 hours, evaluated as a secondary outcome across score-defined risk strata rather than as a model predictor.

The study follows a structured 2-phase framework.

#### Phase I: Prediction Model Development and Internal Validation

Phase I is a nested case-control study within a retrospective cohort of adult patients with FN between December 2021 and December 2024. We will analyze demographic, clinical, laboratory, and physiological variables using multivariable conditional logistic regression to develop the sepsis prediction model, with internal validation by bootstrap resampling (1000 iterations).

#### Phase II: Temporal Validation and Comparative Diagnostic Performance

Phase II is a prospective, same-center temporal validation cohort. Consecutive adult patients with FN will be enrolled, and the bedside nurse formally assigned to each patient’s care will apply the nurse-led score at the onset of FN. A nested substudy, in which additional trained ward nurses on duty independently score the same patients, will quantify interrater agreement and bedside feasibility, and outcome adjudication will be performed separately by assessors blinded to all scores. We will assess the diagnostic performance of the new nurse-led score and compare it with established tools (MASCC, CISNE, NEWS-2, and qSOFA) to evaluate discrimination and clinical usefulness in routine practice. This 2-phase design allows for stepwise model development, temporal validation, and comparative evaluation under real-world hematology and bone marrow transplantation conditions. Figure 1 presents an overview of the 2-phase study framework.

Reporting will follow TRIPOD-AI (Transparent Reporting of a Multivariable Prediction Model for Individual Prognosis or Diagnosis–Artificial Intelligence) for the prediction model components and Standards for Reporting of Diagnostic Accuracy Studies (STARD) 2015 for the diagnostic performance components; completed checklists will be submitted as supplementary appendices after the full study is completed.

**Figure 1.**
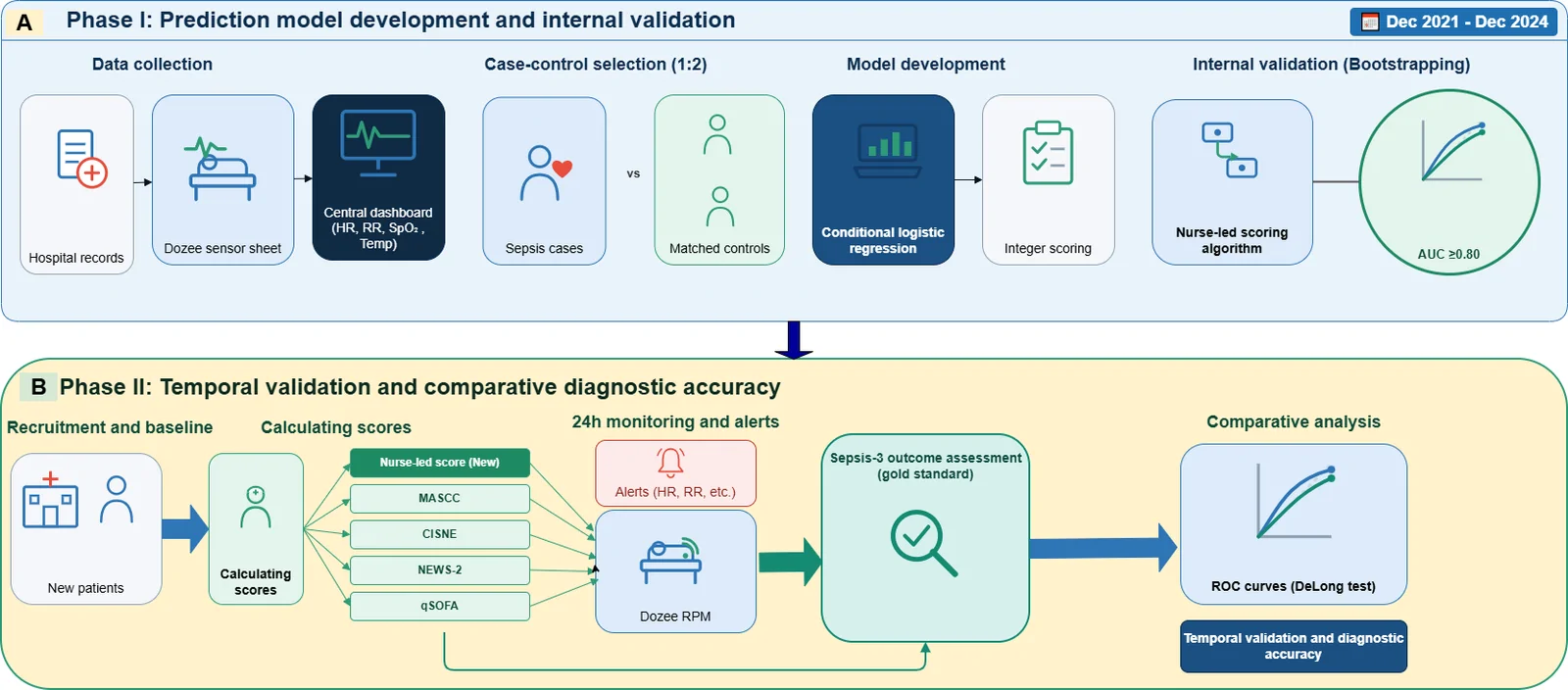
Overview of the nurse-led sepsis scoring framework for adult febrile neutropenia: (A) Phase I (prediction model development and internal validation by nested case-control sampling). Remote patient monitoring (RPM) data collection was continuous throughout the phase I retrospective window (December 2021 onward), consistent with RPM deployment at the study site before cohort entry. (B) Phase II (temporal validation and comparative diagnostic performance assessment against Multinational Association for Supportive Care in Cancer [MASCC], Clinical Index of Stable Febrile Neutropenia [CISNE], National Early Warning Score 2 [NEWS-2], and quick Sequential Organ Failure Assessment [qSOFA]). AUC: area under the receiver operating characteristic curve; HR: heart rate; ROC: receiver operating characteristic; RR: respiratory rate; SpO₂: oxygen saturation.

### Ethical Considerations

This prediction model study and temporal validation protocol is registered with the Clinical Trials Registry-India (registration CTRI/2025/06/088290). The study has received approval from the Institutional Ethics Committee of Amrita School of Medicine (approval ECASM-AIMS-2025‐184). Any substantive modifications to the scoring model, predictor set, or use of AI-enabled RPM data that affect patient safety or study conduct will undergo additional ethics review. This document represents protocol version 2.0, dated September 2026.

### Study Setting

The study will be conducted in the hematology and BMT department at Trustwell Hospital, Bengaluru, India, a multispecialty center that manages patients with hematological malignancies and performs BMT. The hospital has an integrated hospital information system that allows comprehensive retrieval of clinical documentation, laboratory results, and treatment history and uses AI-enabled remote patient monitoring technology that continuously measures vital signs at the bedside using contactless sensors placed under the mattress. The AI-enabled RPM system (Dozee NX-Pro) was implemented as part of routine bedside monitoring in the hematology and BMT unit before December 2021. The retrospective cohort start date (December 2021) was deliberately selected to coincide with RPM availability at the study site, ensuring that contactless, RPM-derived vital sign measurements are consistently available across the entire phase I derivation cohort rather than only for a subset of patients. The hematology and BMT units are staffed by trained oncology nurses experienced in managing FN and noticing early sepsis. This clinical environment provides a suitable platform to evaluate a nurse-led sepsis identification scoring system in high-risk immunocompromised patients.

### Participants

#### Study Population

The study population will include adult patients with hematological malignancies who develop FN during hospitalization or treatment. Patients with leukemia, lymphoma, and multiple myeloma receiving chemotherapy or undergoing hematopoietic stem cell transplantation will be screened for eligibility. This is a highly vulnerable group in whom neutropenia-related infections can progress rapidly to sepsis. We will include only patients who satisfy predefined diagnostic criteria for FN. For phase I, we will construct a retrospective cohort of all eligible patients with FN from December 2021 to December 2024 using the hospital information system. Each patient will contribute only their first episode of FN during the study period, ensuring that no patient is represented more than once in the analysis. From this cohort, we will draw a nested matched case-control sample for prediction model development and internal validation. Phase II will enroll consecutive eligible patients with FN prospectively at the same center.

#### Eligibility Criteria

Participants eligible for inclusion must be aged ≥18 years, have a confirmed diagnosis of a hematological malignancy (leukemia, lymphoma, and multiple myeloma), and be receiving chemotherapy or undergoing hematopoietic stem cell transplantation. Additionally, participants must have developed FN, defined as a single oral temperature ≥38.3 °C or a sustained temperature ≥38.0 °C for at least 1 hour, in the presence of an ANC <500 cells per microliter or an expected decline to <1000 cells per microliter within 48 hours. Patients will be excluded if any of the following criteria are met: (1) simultaneous documentation of FN and sepsis or septic shock at the time of initial clinical assessment, as recorded in the medical records; (2) admission to the ICU before the onset of FN; or (3) incomplete medical records that preclude extraction of key predictor variables.

### Two-Phase Study Framework

#### Phase I: Prediction Model Development and Internal Validation

Phase I will identify sepsis predictors in patients with FN and develop the nurse-led scoring model using a nested case-control design within a retrospective cohort. The researcher will extract retrospective data from the hospital information system for December 2021 to December 2024 to construct a cohort of all eligible patients with FN. We will include only the first FN event per patient, so each patient contributes a single observation. From this cohort, we will draw a nested matched case-control sample: patients who progress to Sepsis-3 sepsis within 14 days of FN onset will be classified as cases. For each case, we will select up to 2 control patients without sepsis within this window in a 1:2 ratio. Controls will be matched on age (caliper +5 or −5 years), gender, type of hematological malignancy, and treatment modality. These matching factors will be treated as design variables and will not be included as candidate predictors, so that the model focuses on acute clinical and physiological elements. For each case, the researcher will randomly select matched controls from the pool of eligible patients in the retrospective cohort. Candidate predictors will include demographic, clinical, laboratory, and physiological variables obtained from medical records and remote monitoring. The researcher will fit a multivariable conditional logistic regression model to identify independent predictors of sepsis, as described in Sepsis-3, in the matched nested case-control sample. The nested matched case-control sampling from the full FN cohort is used exclusively as an efficient design strategy; by applying conditional logistic regression, the study can obtain unbiased estimates of predictor-outcome associations valid for the underlying cohort. We will then convert the regression coefficients from the final model into a simple bedside score: each nonzero coefficient will be divided by the smallest nonzero coefficient, rounded to the nearest whole number, and negative values set to zero, so higher scores reflect higher sepsis risk.

We will calculate the patient’s total score by summing these item weights. This simple scaling approach has been widely used in bedside clinical scores to preserve the relative effect sizes of predictors while producing integer weights that nurses can apply without calculators. We will first evaluate the total score as a continuous predictor using receiver operating characteristic curve (ROC) curve analysis, reporting the area under the ROC (AUC; c-index) with its 95% CI. We will perform internal validation by bootstrap resampling with 1000 iterations to examine model stability and obtain optimism-corrected measures of discrimination.

#### Phase II: Temporal Validation and Comparative Diagnostic Performance

Phase II will evaluate the clinical performance of the scoring system through prospective validation in the same center and a comparative assessment of diagnostic performance. Eligible patients with FN will be enrolled consecutively in a prospective cohort to minimize selection bias. Before enrollment, eligible patients or their legally authorized representatives, where applicable, will be approached by the principal investigator, provided with a participant information sheet, and asked to provide written informed consent in the participant’s preferred language. The phase II validation cohort will be entirely distinct from the retrospective phase I derivation cohort, with no patient overlap, so that validation mimics future real-world use of the score in newly presenting patients. At the onset of FN, we will record baseline demographic, clinical, and laboratory variables required for score calculation and apply the nurse-led scoring system to classify patients into baseline risk categories. At the same time, we will calculate MASCC, CISNE, NEWS-2, and qSOFA scores for each patient to allow direct comparison. During this validation phase, the primary nurse-led score for each participant is calculated at the bedside by the staff nurse formally assigned to that patient’s care at the onset of FN, following the predefined scoring manual and standard operating procedure (Multimedia Appendix 1). Where additional trained ward nurses are on duty and available, they independently calculate the score for the same patient solely for the nested interrater reliability substudy; their scores do not contribute to the primary phase II diagnostic performance analysis. Different individuals perform index test scoring and reference standard adjudication, and no individual performs both functions for the same participant. The principal investigator calculates comparator scores (MASCC, CISNE, NEWS-2, and qSOFA) in parallel from the recorded variables and does not participate in outcome adjudication; because these comparators are established instruments not under evaluation for bedside usability, they are not included in the interrater substudy. All index test scores from the primary rater and, where applicable, from additional substudy raters are entered into a date- and time-stamped study record within 6 hours of FN onset. Entries are locked on submission and cannot be edited, so scoring is completed well before the 14-day outcome window has elapsed and cannot be revised based on the outcome.

### Nested Interrater Reliability and Bedside Feasibility Substudy (Phase II)

Phase II incorporates a nested interrater reliability and feasibility substudy, as the score is intended for independent bedside application by ward nurses. Three rostered staff nurses of the hematology and BMT unit, selected to span the range of unit experience present on the ward, are trained in a single standardized session using the scoring manual (Multimedia Appendix 1) and 5 written case vignettes. They must meet a predefined competency threshold on the vignettes before participating. For each eligible patient enrolled in phase II, the staff nurse formally assigned to that patient’s care at FN onset calculates the score that feeds the primary phase II diagnostic accuracy analysis. Where at least one additional trained nurse is also on duty and available, that nurse (and a third, where rostering permits) independently calculates the nurse-led score for the same patient within the same 60-minute window, while remaining blinded to the primary rater’s and any other rater’s score sheets; these additional scores are used solely to compute interrater agreement and feasibility for this nested substudy and do not contribute to the primary phase II diagnostic accuracy analysis. Completed sheets are sealed upon submission, and raters may not reconcile or discuss results. As ward rostering will not always place all 3 nurses on the same shift, the number of raters per patient varies between 2 and 3; the agreement statistics used accommodate a variable number of raters per subject, and the achieved number of multiply-rated patients, together with the number rated by 3 and by 2 nurses, will be reported rather than fixed in advance. As multiple-nurse bedside scoring cannot be reconstructed retrospectively, the substudy is restricted to patients enrolled from the date of the protocol amendment onward; we therefore report separately the number of patients participating in the substudy and the number participating in the main validation analysis. Agreement will be quantified using Fleiss κ for the binary low- versus high-risk classification and, for each score item, the intraclass correlation coefficient (2-way random effects and absolute agreement) for the total score, exact percentage agreement, as well as pairwise Cohen κ for each rater dyad, each with 95% CIs and interpreted against conventional agreement bands. Feasibility will be assessed as time to complete the score, the proportion of sheets with any missing item and the missing rate per item, and the proportion of patients scored within the specified window. Item-level Fleiss κ below 0.60 will prompt rewording or reoperationalization of that item in the final scoring manual, reported transparently as a protocol-specified refinement. This substudy is noninterventional, does not alter patient management, and imposes no additional patient burden beyond parameters obtained in routine care; participating nurses provide written consent for the collection of their scoring data.

### Data Collection and Measurements

Vital signs will be monitored continuously using the Dozee NX-Pro remote patient monitoring system, which combines a contactless undermattress sensor (ballistocardiography based) to derive heart rate and respiratory rate and connected devices and plug-in probes to capture blood pressure, oxygen saturation, and body temperature. As RPM was already in routine clinical use at the study site before the start of the retrospective cohort period, the same contactless measurement approach captured physiological predictor variables for all phase I patients, avoiding a mixed-methodology dataset combining RPM-derived and manually charted vitals.

#### Phase I

When the first patient with FN is diagnosed, the researcher will retrospectively identify, from the hospital information system, contactless RPM-derived physiological readings (heart rate, respiratory rate, blood pressure, oxygen saturation, and temperature), corresponding laboratory values, and documented clinical signs and symptoms. We will abstract these single point-in-time physiological readings from device-generated entries and nurses’ notes recorded in routine clinical practice and use them as candidate predictors, as described in the “Study Variables” section. In this phase, we will collect additional data on clinical variables (symptoms, infection site, and comorbidities), treatment variables (chemotherapy regimens, antimicrobial therapy, and supportive measures), and clinical outcomes (ICU admission, organ support, length of stay, and in-hospital mortality), along with routine laboratory results.

#### Phase II

We will prospectively follow eligible patients. Each patient will contribute only their first eligible FN episode during the phase II recruitment period. At the onset of FN, we will record baseline demographic, clinical, and laboratory variables required to calculate the new nurse-led score and comparator scores (MASCC, CISNE, NEWS-2, and qSOFA). The predesignated primary rater will calculate the nurse-led score used in the primary phase II analysis for each patient; when additional trained nurses are on duty, they will independently calculate the score for the nested interrater substudy only, following the same procedure described in the “Two-Phase Study Framework” section. All completed score sheets, from the primary rater and any additional raters, will be submitted within 6 hours of FN onset and locked after submission. The principal investigator will separately calculate the comparator scores from the recorded variables and will not participate in primary outcome adjudication. Clinically important RPM alerts that meet the same study-defined thresholds and occur within the first 24 hours after diagnosis of FN will be captured prospectively as present or absent, along with corresponding laboratory results. Simple temperature rises without accompanying hemodynamic or respiratory changes, and brief, nonsustained abnormalities will not be counted as alerts. In phase II, the researcher will collect only the predictor variables needed for the new score and comparator tools, first-24-hour RPM alert status, feasibility and interrater reliability data, and outcome variables for research purposes, beginning after written informed consent. The researcher will extract phase II data from the hospital information system, nursing documentation, and completed bedside score sheets into a standard analysis dataset. The nurse-led score will be obtained prospectively from the independently completed bedside score sheets, whereas the comparator scores will be calculated from the recorded variables using prespecified scoring rules. Multimedia Appendix 1 provides full scoring details and the standard operating procedure.

### Study Variables

#### Predictor Variables

The study will collect demographic, clinical, laboratory, treatment, physiological, and outcome variables, as outlined below. Multimedia Appendix 2 provides detailed definitions, coding schemes, units of measurement, and data collection time windows.

#### Clinical Variables

These will include presenting symptoms, infection site, microbiological findings, and comorbid conditions. The researcher will document symptoms such as fever, chills, fatigue, and other infection-related features. The researcher will identify the anatomical site of infection (eg, respiratory, urinary, bloodstream, or gastrointestinal) based on clinical assessment and investigations. Microbiological findings will include organisms grown in culture. The researcher will record comorbidities such as diabetes mellitus, hypertension, cardiovascular disease, and other chronic illnesses to assess their potential influence on infection severity and treatment response.

#### Laboratory Variables

Laboratory variables will include white blood cell count, ANC, organ function tests, inflammatory biomarkers, and culture results. Routine blood tests will provide hematological markers such as total white blood cell count and ANC. Organ function markers, including renal and hepatic function tests, will be recorded to describe the patient’s physiological status. Inflammatory biomarkers such as C-reactive protein and procalcitonin will be recorded when available because they indicate the strength of the host response to infection. Microbiological culture results will be used to demonstrate infection and, when positive, to identify the organisms involved.

#### Treatment Variables

Treatment-related variables will cover key aspects of anticancer and anti-infective care, as well as important supportive measures. We will document chemotherapy details, including the drugs used, dosing schedules, and treatment cycles. We will document antibiotic therapy, including the specific agents prescribed, treatment duration, and any notable changes or escalations to the antimicrobial plan.

#### Physiological Variables (RPM Derived)

In phase I, physiological variables from the Dozee NX-Pro system—heart rate, respiratory rate, blood pressure, oxygen saturation, and temperature—are captured as single point-in-time readings at FN onset and used as candidate predictors, as described in the “Data Collection and Measurements” section. In phase II, the same system also continuously monitors to detect clinically significant RPM alerts within the first 24 hours, which are considered a secondary outcome (see “Outcome” section).

#### Clinical Outcome Variables

We will collect clinical outcomes to describe the overall course and severity of the disease. These will include the total length of hospital stay, need for ICU admission, and any requirement for advanced organ support, such as mechanical ventilation or renal replacement therapy. In-hospital mortality will be recorded as a key end point. Length of stay will be calculated by the researcher from the date of admission to the date of discharge. ICU admission and the requirement for advanced organ support will be recorded as markers of disease severity.

### Outcome

The primary outcome is sepsis in patients with FN, defined using Sepsis-3 as suspected or confirmed infection with an increase of at least two points in the Sequential Organ Failure Assessment (SOFA) score from baseline. Baseline SOFA will be reconstructed from clinical data recorded before or at the onset of FN, with chronic organ dysfunction (eg, preexisting renal or respiratory insufficiency) reflected in the baseline score, so only acute worsening contributes to the required ≥2-point increase.

In both phase I and phase II, the primary outcome is Sepsis-3 sepsis occurring within 14 days of the onset of FN, ensuring a consistent outcome window for model derivation and temporal validation. This 14-day outcome window was adopted via a protocol amendment in September 2026 to align the Phase I and Phase II outcome definitions; the original Phase I outcome definition (Sepsis-3 sepsis at any point during the index admission) and the chronology of this change relative to Phase I model derivation are described in the “Results” section. In phase I, we retrospectively reconstruct SOFA scores from routinely recorded respiratory, cardiovascular, hepatic, renal, hematologic, and neurologic data and compare the calendar date patients first met Sepsis-3 criteria with the date of FN onset to confirm the event falls within the 14-day window. In phase II, we prospectively collect SOFA components from clinical records and monitoring systems within the same window. Patients who meet Sepsis-3 criteria more than 14 days after FN onset are classified as nonevents for the primary outcome in both phases, consistent with the study’s focus on early sepsis detection at the point of greatest clinical actionability.

### Outcome Ascertainment and Adjudication

When full SOFA reconstruction is not possible, we will apply predefined Sepsis-3–aligned surrogate organ dysfunction criteria (Multimedia Appendix 3). In phase I, 2 adjudicators independent of the clinicians who treated the patients will review each retrospective patient with FN independently, using a structured data abstraction sheet and the predefined adjudication algorithm described in Multimedia Appendix 3. Both adjudicators will be blinded to the study’s candidate predictor variables when recorded separately from outcome-relevant data; however, because predictor values and SOFA components are embedded within the same retrospective clinical record, complete blinding to all predictor information cannot be guaranteed for every patient. This represents a limitation of retrospective outcome ascertainment and is discussed later on. A third adjudicator or senior clinician will resolve disagreements between the 2 adjudicators, and interrater agreement for the binary sepsis classification will be assessed using Cohen κ coefficient.

In phase II, 2 assessors independently adjudicate the primary sepsis outcome: a clinician not involved in the patient’s clinical care and a trained research adjudicator, applying the same predefined Sepsis-3–aligned algorithm and structured abstraction sheet used in phase I (Multimedia Appendix 3). Neither adjudicator participates in bedside scoring. Both are blinded to all nurse-led and comparator score values: completed score sheets are stored in a separate, access-restricted file, and adjudicators receive a deidentified clinical extract from which all score sheets, score values, and risk category labels have been removed. Adjudication occurs after the 14-day outcome window has closed, using the locked score record described earlier so that neither assessment can influence the other. Disagreements are resolved by a third senior clinician who is likewise blinded to score values, and interrater agreement for the binary sepsis classification is quantified using Cohen κ with 95% CIs. Phase II therefore conforms to STARD 2015 items 13a and 13b for blinded, independent reference standard assessment. As phase II candidate predictors and SOFA components come from the same clinical record, we cannot entirely exclude residual incorporation bias; we discuss this in the “Limitations” section.

### Sample Size Considerations

#### Phase I (Prediction Model Development and Internal Validation)

For phase I scoring system development, we will use a retrospective cohort of approximately 140 adult patients diagnosed with FN from December 2021 to December 2024 at our institution, with an anticipated 40 sepsis events (defined as Sepsis-3 sepsis occurring within 14 days of FN onset, consistent with the phase II outcome window) based on audit data. All available sepsis patients will be included as cases, together with about 80 nonsepsis patients sampled from the remaining cohort to achieve an efficient 1:2 case-to-control ratio in a nested matched case-control study, in line with recommended matching strategies to improve efficiency in case-control and prognostic model studies [31]. In accordance with contemporary guidance for clinical prediction models and TRIPOD recommendations, we evaluate sample size adequacy primarily by the number of outcome events rather than total sample size, and we include all eligible data within the feasible time window to maximize informational value [32,33]. We anticipate that the final nurse-led sepsis score will contain 6 to 8 predictors; with approximately 40 events, this corresponds to approximately 5 to 7 events per predictor parameter, which is below traditional “10 events per variable” rules but considered acceptable when combined with parsimonious predictor preselection and appropriate internal validation [34]. To limit overfitting, we will therefore restrict predictors to those with a strong clinical rationale and prior evidence and perform internal validation by bootstrapping to quantify and adjust for optimism in model effectiveness, consistent with recent recommendations for prediction model development in modest sample sizes [35]. Given the anticipated 5 to 7 events per predictor parameter, we will perform a post hoc uniform coefficient shrinkage sensitivity analysis, in addition to bootstrap-based internal validation, to quantify and, where indicated, correct for optimism arising from the modest events-per-variable ratio [36,37]. Conditional logistic regression is appropriate for this matched nested case-control design and yields unbiased association estimates that researchers can translate into prediction scores [38]. The structured candidate predictor variables will include comorbidity profile, neutropenia, and infection course characteristics available at the moment of scoring, baseline same-day laboratory findings, and bedside physiological parameters, as detailed in Multimedia Appendix 2. Age, gender, underlying hematological malignancy, and treatment modality are matching variables in the phase I nested case-control design and are not included as candidate predictors; microbiological and treatment response variables that are not available at the moment of scoring and clinical outcome variables are collected for descriptive and outcome analysis but are similarly excluded from the candidate predictor set. We will evaluate the model’s discriminative performance (ROC-AUC) with internal validation via bootstrapping.

#### Phase II (Temporal Validation and Diagnostic Performance)

For phase II temporal validation, the main objective is to obtain reasonably precise estimates of discrimination (AUC) and to provide exploratory comparative performance data relative to established tools (MASCC, CISNE, NEWS-2, and qSOFA), rather than to be formally powered to detect small superiority margins in AUC. Given the anticipated phase II sample size, calibration assessment (calibration plot, calibration-in-the-large, and calibration slope) is intended to provide a descriptive, clinically interpretable picture of agreement between predicted and observed risk, rather than to be formally powered for a specific calibration-slope precision target; CIs around calibration estimates will be reported to convey statistical variability. The validation cohort will therefore include all eligible consecutive patients with FN during the recruitment period, with the final sample size and number of sepsis events determined by case availability and feasibility. We will report CIs for AUC along with other performance measures to reflect statistical variability. We anticipate that including all consecutive patients with FN during the recruitment period will yield sufficient events to estimate the AUC with CIs of approximately ±0.05‐0.10. However, the actual precision will depend on the observed sepsis event rate. If temporal validation shows poor discrimination (eg, AUC <0.70) or systematic miscalibration, we will consider exploratory model updating, as described in the “Statistical Analysis” section [39,40]. For the nested interrater reliability substudy, we will not present a formal sample size calculation because the objective is a descriptive estimate of agreement rather than hypothesis testing. We assess agreement among all eligible phase II patients scored by 2 or more of the 3 trained ward nurses. We will report the number of multiply-rated patients, the number of raters per patient, and CIs around all agreement estimates. Hence, the precision actually attained is transparent rather than assumed in advance.

### Statistical Analysis

#### Overview

Data analysis will commence by assessing dataset completeness and patterns of missing values. If fewer than 5% of values are missing for key predictors, complete case analyses will be used; if missingness is more frequent and appears to be missing at random, multiple imputation by chained equations will be used in phase I to impute important predictors such as ANC, procalcitonin, and white blood cell count, using an imputation model that includes all candidate predictors and the outcome. The researcher will create 20 imputed datasets, and phase I estimates will be combined using Rubin’s rules. Missing data imputation will be used only for phase I model development; phase II model effectiveness will be evaluated primarily using complete-case analyses, with sensitivity analyses if missingness is nontrivial. We will not include predictors with more than approximately 40% missing values. Baseline characteristics will be summarized using means and SDs or medians and IQRs for continuous variables and frequencies and percentages for categorical variables.

#### Phase I (Prediction Model Development and Internal Validation)

For the main objective, a set of clinically and biologically plausible predictors, selected a priori from FN and sepsis literature and expert opinion, will be entered into a multivariable conditional logistic regression model within the matched nested case-control sample. Continuous predictors will be recorded as measured but entered either as linear terms or as prespecified clinically meaningful categories to represent nonlinear risk relationships without excessively complex functional forms. Univariate tests (*t* tests or Mann-Whitney *U* tests for continuous variables and chi-square or Fisher exact tests for categorical variables) will describe crude associations but will not, on their own, determine variable selection. We will retain clinically important prespecified predictors even if univariate *P* values are not statistically significant. At the same time, we may remove clearly uninformative variables to limit overfitting, given the modest number of outcome events. The primary model will not include interaction terms; we examine exploratory interactions only in secondary, hypothesis-generating analyses. We will not use penalized regression methods, such as least absolute shrinkage and selection operator; instead, we will focus on a prespecified set of predictors and a transparent conditional logistic regression model. As a sensitivity analysis to further address potential overfitting beyond standard bootstrap-based optimism correction, we will evaluate post hoc uniform coefficient shrinkage. We will base the shrinkage on the bootstrap-derived, optimism-corrected calibration slope from the 1000 bootstrap resamples. We will retain conditional logistic regression as the primary model for coefficient evaluation. We will report the shrinkage factor alongside the unshrunk coefficients to characterize the degree of optimism in apparent model classification. If this analysis shows limited evidence of coefficient overfitting, the existing regression coefficients will be retained in the final bedside score to preserve interpretability for nurse-led application; if more substantial shrinkage is indicated, shrunk coefficients will be used instead, and any such adjustment will be reported transparently as a post hoc modification.

As some provisional score items under consideration, including measures related to blood pressure, oxygen saturation, and respiratory rate, are physiologically related to the cardiovascular and respiratory components of the SOFA score used to define the primary outcome, and because phase I predictors and reconstructed SOFA components are derived from the same clinical records, there is a risk that this coincidence could increase apparent model effectiveness. To address this, if any of the final selected predictors in the fitted model conceptually overlap with SOFA’s cardiovascular or respiratory domains, or with qSOFA criteria, a prespecified sensitivity analysis will be conducted: the model will be refit with these overlapping items removed, and the resulting AUC will be reported alongside the AUC of the full model to quantify the extent of this potential inflation.

We will internally validate the prediction model using 1000 bootstrap resamples. We will assess discrimination using the AUC and obtain optimism-adjusted AUC estimates with 95% CIs via bootstrapping to quantify overfitting and estimate expected performance in similar patient populations. We will report agreement between the 2 phase I outcome adjudicators as Cohen κ with 95% CIs, along with the proportion of episodes requiring third-adjudicator resolution.

#### Phase II (Temporal Validation and Diagnostic Validity)

In phase II, we will evaluate the nurse-led score’s discrimination using AUC, along with standard diagnostic performance measures, such as sensitivity, specificity, positive predictive value, and negative predictive value. We finalized the phase II statistical analysis plan as part of the protocol amendment described earlier. Although enrollment commenced in December 2025, we have not yet performed discrimination, calibration, or comparative accuracy analyses of the phase II dataset. Two independent blinded assessors will readjudicate previously enrolled patients, and we will lock the analysis dataset at the close of recruitment before any diagnostic performance analysis. Any later analysis not specified here will be reported as post hoc. We will report agreement between the 2 phase II outcome adjudicators as Cohen κ with 95% CIs, along with the proportion of patients requiring third-adjudicator resolution. The primary analysis will use the consensus outcome, with a sensitivity analysis restricted to patients on whom both adjudicators agreed without third-party resolution. For the nested substudy, interrater agreement among ward nurses will be summarized using Fleiss κ for the binary risk classification and each score item, the intraclass correlation coefficient for the total score, and raw percentage agreement, all with 95% CIs. As the number of raters per patient may vary between 2 and 3 depending on ward rostering, agreement analyses will accommodate a variable number of raters. Feasibility measures will be summarized as means with SDs as well as medians with IQRs, and as proportions. The diagnostic accuracy analysis, including AUC, calibration, sensitivity, specificity, positive predictive value, and negative predictive value, will use the score assigned by the predesignated primary rater (ie, the bedside nurse formally responsible for the patient’s care at FN onset) for each participant. This is the sole basis for phase II diagnostic performance and risk classification; scores from additional substudy raters are not used in discrimination, calibration, or accuracy estimation and instead contribute exclusively to the interrater agreement and feasibility analyses described earlier. Calibration of the nurse-led score will be evaluated using a calibration plot of observed versus predicted sepsis probability across deciles of predicted risk, together with the calibration intercept (calibration-in-the-large, reflecting whether predicted risk corresponds to the overall observed sepsis rate) and the calibration slope (reflecting whether predicted risk is appropriately spread across the range of observed risk, with a slope of 1 indicating ideal calibration). Calibration will not be formally assessed in phase I because the nested matched case-control sampling design used for model derivation artificially sets the case-to-control ratio and does not preserve the natural sepsis event rate of the underlying cohort, making calibration-in-the-large uninterpretable without a sampling-fraction correction that is not planned in this protocol; phase I therefore focuses on discrimination (AUC) and internal validation by bootstrap resampling, consistent with standard practice for case-control–derived prediction models. We will compare the score with MASCC, CISNE, NEWS-2, and qSOFA using the DeLong test for AUC plus the McNemar test for paired sensitivity and specificity. Phase II primarily aims to obtain precise estimates of AUC, calibration, and comparative performance, rather than formally test small differences between tools.

### Model Updating and Exploratory Analyses

If temporal validation shows poor discrimination or systematic miscalibration, model updating may include recalibrating intercept and slope, re-estimating selected coefficients, or adjusting score cutoffs to improve clinical usefulness. We will clearly report any changes as post hoc updates and consider them preliminary until validated in future studies. We will examine associations between baseline score–defined risk groups and RPM alerts using chi-square tests, and estimate odds ratios with 95% CIs for higher- versus lower-risk groups as exploratory analyses. All statistical analyses will be performed using IBM SPSS (version 29.0) and Python (version 3.13); Python code used for procedures such as bootstrap resampling will be archived to support reproducibility and transparency of the modeling process.

### Ethical Considerations

The study protocol received approval from the Institutional Ethics Committee (IEC) of Amrita School of Medicine (registration ECNEW/INST/2023/KL/0379) on April 16, 2025 (approval ECASM-AIMS-2025‐184). Trustwell Hospital, Bengaluru, the designated site for recruitment and data collection, does not maintain its own institutional ethics committee. The IEC formally reviewed and noted the institutional permission letter from Trustwell Hospital as part of its assessment before granting ethical clearance, hence extending ethics oversight to this site. In addition, the principal investigator obtained separate institutional permissions authorizing data access and study conduct from the administration of Trustwell Hospital (dated March 11, 2025) and from the Director of the Bone Marrow Transplantation and Hematology Department at Trustwell Hospital. All prospective participants will provide written informed consent before enrollment in phase II, and the participant information sheet and informed consent form are provided as Multimedia Appendix 4. The study will maintain patient confidentiality by anonymizing all extracted clinical data and restricting access to the study database to authorized research personnel; it will follow the Declaration of Helsinki and institutional research governance guidelines. The AI-enabled system (Dozee NX-Pro) will store remote patient monitoring data on secure institutional computers, with access permissions and audit trails in accordance with hospital policy. The RPM system will be used as a source of contactless point-in-time physiological predictor data in phase I and as a source of continuous monitoring–derived alert data for the phase II secondary outcome; in neither phase will it independently initiate or modify treatment, and clinical responsibility will remain entirely with the treating hematology and bone marrow transplantation teams. We will document and report phase II to the ethics committee in accordance with institutional procedures. As this manuscript reports a study protocol and does not include any person’s identifiable data (including images or videos), specific patient consent for publication is not applicable.

## Results

Phase I retrospective data extraction and model development proceeded in 2 stages. An initial extraction and model derivation, using Sepsis-3 sepsis at any point during the index admission as the original primary outcome, commenced in May 2025 and was completed in November 2025.

During protocol revision in September 2026, we specified 3 analytical decisions before any rederivation was undertaken: (1) alignment of the phase I and phase II primary outcome to a consistent 14-day window; (2) a sensitivity analysis refitting the model with predictors that conceptually overlap the SOFA cardiovascular and respiratory domains removed; and (3) a post hoc uniform coefficient shrinkage analysis. We specified all 3 in advance of the rederivation to which they apply. We are therefore rederiving the phase I model under the revised 14-day outcome definition; this rederivation is in progress, and this protocol reports no phase I model coefficients, discrimination estimates, or risk category cutoffs. The November 2025 derivation under the first outcome definition is superseded and will not be reported as a leading result. As part of outcome ascertainment, we will report the proportion of retrospectively identified Sepsis-3 events occurring after day 14 of the index admission alongside the rederived phase I model to document the extent to which aligning the phase I and phase II outcome windows (see “Outcome” section) affects the composition of the derivation cohort.

Phase II prospective temporal validation commenced in December 2025. As of September 2026, 22 participants had been enrolled, and enrollment and data collection were ongoing, with completion expected in December 2026. Following the September 2026 protocol amendment, phase II incorporates bedside scoring by trained frontline ward nurses, a nested interrater reliability and feasibility substudy, and outcome adjudication by 2 independent assessors blinded to all score values. Before the amendment, the principal investigator prospectively recorded the nurse-led and comparator scores. Following the amendment, primary outcomes for all phase II participants, including those enrolled before the amendment, are being readjudicated by the 2 independent blinded assessors to apply a uniform blinded reference standard across the validation cohort. As of September 2026, no discrimination, calibration, or comparative diagnostic performance analyses of the phase II dataset had been performed. Outcome readjudication will be completed, and the analysis dataset locked before these analyses are undertaken. We expect to submit the main study results, including the rederived phase I model and the phase II temporal validation and comparative diagnostic performance findings, for publication in 2027.

## Discussion

### Principal Results

This protocol addresses an important unmet need in early sepsis identification among adults with FN by proposing a context-specific, nurse-led predictive scoring system embedded within hemato-oncology and BMT supportive care. Existing tools provide general risk stratification but have limited sensitivity and discrimination in immunocompromised patients with sepsis, pointing to the need for adapted models in this group [41,42]. The planned score aims to bridge this gap by combining FN–specific clinical and laboratory variables with precisely measured, contactless physiological parameters captured at the onset of FN, so risk prediction better represents the unique pathobiology and rapid clinical course of this population. Conceptually, the score is designed not as a unit-specific instrument but as a structured supportive oncology tool that can guide nurse-led escalation pathways, early sepsis workup, and treatment continuity in high-risk hematology patients.

A key strength of this protocol is its methodological strictness. The nested matched case-control design in phase I allows efficient identification of predictors while controlling for key design variables, such as malignancy type and treatment modality, consistent with recommendations for matched case-control prognostic studies [43,44]. Multivariable conditional logistic regression, followed by transforming regression coefficients into an integer-weighted bedside score, balances statistical strength with bedside usability. Internal validation by bootstrap resampling and a distinct same-center temporal validation cohort align with best practice for prediction-model development and validation, which emphasizes appropriate internal, internal-external, and external validation to avoid overfitting and maintain transportability [45].

The protocol also uses contactless remote patient monitoring to capture accurate point-in-time physiological predictors in phase I, while additionally examining continuous monitoring–derived alerts as a secondary marker of physiological instability in phase II. Continuous assessment has been shown to detect subtle physiologic changes that precede clinically apparent deterioration more effectively than intermittent vital sign checks [46,47]. Remote monitoring equipped with early-warning systems has improved ward safety and the timeliness of escalations in tertiary care hospital wards. By examining first-24-hour RPM alerts alongside clinical and laboratory data, this study may clarify temporal patterns of physiological instability in FN and enhance the granularity of sepsis risk stratification. The nurse-led nature of the scoring system, with a PhD nursing scholar and senior BMT-hematology nurse leading design and application, moves decision support closer to the bedside and is intended to reduce variation in clinical judgment, support earlier recognition of deterioration, and improve communication within multidisciplinary teams.

Risk stratification at the onset of FN directly affects supportive oncology care. A high-risk score can trigger earlier empiric antibiotics and hemodynamic optimization, which may prevent progression to septic shock and avoid unplanned ICU transfer. Conversely, clearer stratification may help clinicians decide between safe ward-based management and timely ICU input. At the same time, identifying truly low-risk patients can support maintenance of planned chemotherapy dose intensity and reduce treatment delays due to sepsis-related instability. This score is intended not only as a bedside assessment tool but also as an enabler of structured, nurse-led supportive oncology care, linking real-time physiological surveillance with escalation pathways that can preserve treatment continuity and reduce acute care burden in high-risk hematology patients [48,49]. This aligns with international developments in proactive supportive oncology and early supportive or palliative care interventions but focuses specifically on the underemphasized domain of sepsis-related risk stratification in FN. As the score provides structured risk categories at the onset of FN, it may also facilitate clearer communication between bedside nurses, oncologists, and intensivists, for example, by prompting risk-stratified triggers for early ICU consultation or high-dependency care [15,49].

### Comparison With Prior Work

Risk stratification tools such as the MASCC risk index and the CISNE have been widely used to identify patients with low- and high-risk FN for outpatient versus inpatient management. Prospective validation of the MASCC risk index showed that a score ≥21 could correctly classify most patients with low-risk FN, with high predictive values for an uncomplicated course [50]. However, subsequent systematic reviews and meta-analyses [22] comparing MASCC and CISNE have highlighted important limitations in sensitivity for predicting serious complications, with performance varying by score and threshold. These results show that oncology-specific parameters may not provide sufficient sensitivity to identify early sepsis risk in immunocompromised patients with FN, particularly in settings where acute clinical deterioration is common.

Physiology-based early-warning scores, such as NEWS and Modified Early Warning Score (MEWS), have also been evaluated for FN. In emergency department cohorts, NEWS and MEWS have shown strong prognostic value for early mortality, sometimes outperforming oncology-specific scores such as MASCC and CISNE. [51]. They can be calculated quickly at presentation using vital signs. At the same time, these scores rely on overt physiological abnormalities and established organ dysfunction and were primarily designed for general acute illness rather than FN-specific sepsis. Their focus on short-term mortality and their dependence on intermittent vital-sign measurements may limit their usefulness for detecting the very earliest stages of FN-related sepsis in continuously monitored hematology patients

Several other FN risk scores and prediction models have been developed for specific purposes, such as predicting first-cycle FN risk in patients receiving myelosuppressive chemotherapy or identifying risk factors for multidrug-resistant bacteremia in FN patients. These models have advanced understanding of FN risk and have informed guideline-based recommendations. Still, they frequently focus on chemotherapy cycle-level risk or specific infection outcomes rather than early sepsis detection at the bedside. In addition, most existing FN scores were developed by previous researchers lacking continuous remote monitoring and do not incorporate granular, time-linked physiological data that could capture subtle deterioration before obvious clinical signs appear [52].

From a nursing perspective, oncology nurses have a central role in early identification and management of neutropenic sepsis and in leading protocol-based pathways. Nurse-initiated neutropenic sepsis pathways have been associated with more timely antibiotic administration and more appropriate initial antibiotic selection, supporting the potential value of structured nurse-led care [53]. 54However, few existing FN risk tools are explicitly designed as nurse-led bedside scores that integrate continuous remote patient monitoring data and FN-specific clinical information into a single, interpretable sepsis risk stratification framework

This protocol builds on existing work by bringing together 3 strands: FN-specific risk stratification, physiology-based early-warning scores, and nurse-led sepsis pathways. It extends FN risk scoring beyond unchanging indices by incorporating contactless, accurately measured physiological data from remote monitoring systems as predictor input in phase I, and by examining continuous monitoring–derived alerts as a secondary marker of physiological instability in phase II, while retaining a regression-based, interpretable model that nurses can apply at the bedside. By focusing on adult hematology and BMT patients with FN and embedding the score within nurse-led supportive care and escalation processes, the study addresses a gap in current practice: tailored FN-specific, nurse-centered sepsis prediction models are not yet available [26].

### Limitations

The researcher should acknowledge several limitations of this study design. The retrospective derivation phase depends on the completeness and quality of routine documentation in the hospital information system and nursing notes. Missing data and variation in how clinical signs or RPM alerts are recorded may affect the availability and accuracy of some candidate predictors and SOFA components. The study is conducted in a single tertiary hematology and bone marrow transplantation unit in an urban private hospital in Bengaluru, which may limit generalizability to centers with different patient profiles, chemotherapy protocols, staffing levels, or access to continuous remote monitoring technology. Future work should examine performance in multicenter cohorts, including hospitals without RPM, and may need to develop a version of the score that relies only on routinely observed vital signs. As phase I physiological predictors are derived from single readings at FN onset rather than continuous trend data, the model does not yet exploit the full continuous monitoring capability of the RPM system; incorporating trend- or variability-based features is identified as a direction for future model refinement, informed by the phase II RPM alert analysis.

As candidate predictors and the reconstructed SOFA-based outcome in phase I come from the same clinical records, and some candidate physiological predictors are conceptually related to SOFA’s cardiovascular and respiratory components or to qSOFA criteria, this overlap may inflate apparent model discrimination. We plan a prespecified sensitivity analysis that refits the model without these overlapping predictors and reports both AUCs side by side to quantify this effect (see “Statistical Analysis” section).

Aligning the phase I and phase II primary outcome to a consistent 14-day window improves comparability of model performance across phases but excludes later-occurring sepsis events from the phase I derivation sample; as bone marrow transplantation admissions often extend beyond 14 days, some patients who develop sepsis later in their admission will not contribute a case event to model development, which may limit the model’s applicability to later-onset sepsis and will be examined further in future work.

Outcome adjudication differs in rigor between the 2 phases. In phase I, sepsis outcomes are adjudicated independently by 2 reviewers who are not involved in the patients’ clinical care, with disagreements resolved by a third reviewer and interrater agreement quantified using Cohen κ; however, because candidate predictor values and SOFA components are both recorded within the same retrospective clinical chart, full blinding of adjudicators to predictor data cannot be guaranteed for every patient, and residual incorporation bias cannot be excluded by the researcher. In phase II, separate individuals perform index test scoring and outcome adjudication, and 2 assessors blinded to all score values adjudicate the primary outcome, in line with STARD 2015 recommendations for independent, blinded reference-standard assessment. Two residual limitations remain. First, because phase II predictor values and SOFA components are both documented in the same clinical record, adjudicators may encounter the underlying clinical variables although score values and risk categories are withheld; residual incorporation bias therefore cannot be entirely excluded, and its expected direction is toward modest overestimation of apparent accuracy. Second, adjudicators work within the same clinical unit and cannot be blinded to whether a participant is enrolled in the study.

Restricting the analysis to the first FN event per patient avoids clustering. It simplifies modeling, but it may reduce direct applicability to patients who experience recurrent FN across multiple chemotherapy cycles. The matched case-control design in phase I means variables used for matching are controlled by design and cannot be fully evaluated as independent predictors in the final model. We now evaluate interrater reliability and bedside feasibility of the nurse-led score prospectively in phase II through a nested substudy in which trained frontline nurses independently score the same patients. This substudy has 5 notable limitations. First, the rater pool comprises 3 nurses from a single unit who received identical training, so agreement may be higher than would be observed after routine, less intensive dissemination to a larger and more heterogeneous nursing workforce. Second, the substudy is designed for descriptive precision rather than hypothesis testing, and with 3 raters the CIs around agreement estimates will remain appreciable. Third, ward rostering means some patients will be scored by 2 rather than 3 nurses, which reduces precision for those patients. Fourth, the substudy applies only to patients enrolled after the protocol amendment because bedside scoring by multiple nurses cannot be reconstructed for patients enrolled earlier in phase II; interrater agreement is therefore estimated in a subset of the validation cohort rather than across all enrolled patients. Finally, the substudy measures whether nurses can apply the score consistently and quickly, but not whether doing so changes escalation behavior, workload, alarm fatigue, or patient outcomes; these remain the objectives of a subsequent implementation study.

Finally, same-center temporal validation in phase II provides an important test of performance over time but does not replace formal external validation. Before the principal investigator can recommend widespread adoption, the score will require multicenter external validation and, if needed, recalibration in diverse oncology settings, including centers with different RPM infrastructure and supportive care models.

### Conclusions

This protocol describes a nurse-led clinical prediction model study designed to improve early recognition of sepsis in adults with hematological malignancies who develop FN. By combining FN-specific clinical and laboratory variables with accurately measured, contactless physiological data from remote patient monitoring, and by embedding the score within hemato-oncology and BMT supportive care, the planned model aims to provide more sensitive and timely risk stratification than existing general early-warning or complication scores. The regression-based, interpretable design is intended to make the score usable at the bedside by oncology nurses, supporting earlier escalation, more consistent sepsis workup, and better alignment between continuous physiological surveillance and nurse-led decision-making.

If phase I and phase II confirm good discrimination and calibration and show that the score performs at least as well as MASCC, CISNE, NEWS-2, and qSOFA for FN-related sepsis, the tool could help reduce missed or delayed sepsis recognition, unplanned ICU transfers, and chemotherapy disruption in this high-risk population. However, real-world impact will depend on successful nurse-led implementation, integration with existing sepsis bundles and supportive oncology pathways, and formal multicenter external validation across diverse settings, including centers without advanced remote monitoring capabilities. The longer-term goal is a practical, scalable, nurse-centered bedside score that links continuous monitoring with structured escalation pathways, strengthening supportive care and potentially improving outcomes for hemato-oncology and BMT patients with FN.

## Supplementary material

10.2196/107468 Multimedia Appendix 1 Scoring rules for nurse-led and comparator risk scores.

10.2196/107468 Multimedia Appendix 2 Study variable dictionary for all study phases.

10.2196/107468 Multimedia Appendix 3 Outcome adjudication framework and surrogate sepsis criteria.

10.2196/107468 Multimedia Appendix 4 Informed consent.

## Abbreviations

ANC: absolute neutrophil count
AUC: area under the receiver operating characteristic curve
BMT: bone marrow transplantation
CISNE: Clinical Index of Stable Febrile Neutropenia
FN: febrile neutropenia
ICU: intensive care unit
MASCC: Multinational Association for Supportive Care in Cancer
MEWS: Modified Early Warning Score
NEWS: National Early Warning Score
NEWS-2: National Early Warning Score 2
qSOFA: quick Sequential Organ Failure Assessment
ROC: receiver operating characteristic curve
RPM: remote patient monitoring
SOFA: Sequential Organ Failure Assessment
STARD: Standards for Reporting of Diagnostic Accuracy Studies (STARD 2015)
TRIPOD-AI: Transparent Reporting of a Multivariable Prediction Model for Individual Prognosis or Diagnosis–AI extensions
