# Supplementary material for: Nurse-Led Sepsis Scoring System for Adult Febrile Neutropenia: Protocol for a Two-Phase Clinical Prediction Model Study With Remote Patient Monitoring
Source: JMIR Res Protoc. 2026 Nov 5;15:e107468. doi: 10.2196/107468 (PMC13740224; doi:10.2196/107468)
Supplement: Supplementary file 1 [file resprot-v15-e107468-s002.pdf]

Multimedia Appendix 2

Study variable dictionary for all study phases

This additional file presents a comprehensive inventory of all variables collected across Phase I and Phase II, including candidate predictors, matching variables used in the Phase I case-control design, descriptive/contextual variables, and outcome variables. Variables will be extracted from the hospital information system, laboratory records, nursing records, and microbiology databases, where available. Each variable is labelled in the 'Variable Role' column as Predictor, Matching variable, Descriptive, or Outcome. Only variables labelled 'Predictor' and confirmed to be available at the moment of scoring (at febrile neutropenia onset) are entered as candidate predictors in the nurse-led score. Laboratory values are same-day results obtained at febrile neutropenia onset in this inpatient setting and are therefore available at the moment of scoring

Variable dictionary

Domain	Variable	Operational definition/unit	Planned time window	Variable Role
Demographic	Age	Age in completed years at onset of febrile neutropenia; used as a Phase I matching variable, with cases and controls matched within a caliper of ±5 years (maximum allowable age difference of 5 years)	At febrile neutropenia onset	Matching variable (Phase I, caliper ±5 years); not a candidate predictor
Demographic	Gender	Male = 1; Female = 0	Baseline at febrile neutropenia onset	Matching variable (Phase I); not a candidate predictor
Comorbidity	Comorbidity pattern	Coded categories for diabetes, hypertension, hypothyroidism, and their combinations	Baseline at febrile neutropenia onset	Predictor

Comorbidity	Number of comorbidities	Dichotomized as fewer than 2 versus 2 or more comorbidities	Baseline at febrile neutropenia onset	Predictor
Disease-related	Haematological malignancy type	Leukaemia, lymphoma, multiple myeloma, or other haematological malignancy	Baseline at febrile neutropenia onset	Matching variable (Phase I); not a candidate predictor
Disease-related	Treatment modality	High-dose chemotherapy for leukaemia, haematopoietic stem cell transplantation, or chemotherapy for lymphoma or multiple myeloma	Baseline or index treatment	Matching variable (Phase I); not a candidate predictor
Neutropenia or infection course	Dates of diagnosis, admission, treatment start, ANC below 500, and febrile neutropenia onset	Calendar dates used to derive time intervals in days	Historical or index admission	Descriptive
Neutropenia or infection course	Duration of neutropenia	Number of days with ANC below 500; dichotomized as 7 days or less versus more than 7 days	During index febrile neutropenia episode	Descriptive; not available at moment of scoring
Neutropenia or infection course	Clinical site of infection	Blood, urine, lungs, abdomen, PICC line, perianal, multiple sites, or no definite site identified	During index febrile neutropenia episode	Descriptive; not fully available at moment of scoring

Neutropenia or infection course	Number of clinical infection sites	Fewer than 2 sites versus 2 or more sites	During index febrile neutropenia episode	Descriptive; not fully available at moment of scoring
Laboratory	Haemoglobin	g/dL	Closest same-day value at febrile neutropenia onset	Predictor
Laboratory	Total white blood cell count	Cells/µL	Closest same-day value at febrile neutropenia onset	Predictor
Laboratory	Absolute neutrophil count	Cells/µL	Closest same-day value at febrile neutropenia onset	Predictor
Laboratory	Platelet count	Cells/µL	Closest same-day value at febrile neutropenia onset	Predictor
Laboratory	Serum creatinine	mg/dL	Closest same-day value at febrile neutropenia onset	Predictor

Laboratory	Total bilirubin	mg/dL	Closest same-day value at febrile neutropenia onset	Predictor
Laboratory	Procalcitonin	ng/mL; analysed as continuous and, if required, as categories	Closest same-day value at febrile neutropenia onset	Predictor
Radiology	Lung infiltrates on imaging	Presence of infiltrates on chest radiograph or CT chest (Yes = 1; No = 0)	At or within 24 hours of febrile neutropenia onset	Descriptive; not always available at moment of scoring
Physiological	Temperature	°C	At febrile neutropenia onset	Predictor
Physiological	Heart rate	Beats/minute	At febrile neutropenia onset	Predictor
Physiological	Respiratory rate	Breaths/minute	At febrile neutropenia onset	Predictor
Physiological	Systolic blood pressure	mmHg	At febrile neutropenia onset	Predictor
Physiological	Diastolic blood pressure	mmHg	At febrile neutropenia onset	Predictor

Physiological	Oxygen saturation	SpO2 (%)	At febrile neutropenia onset	Predictor
Physiological	Glasgow Coma Scale	Total score	At febrile neutropenia onset	Predictor
Physiological	Clinical symptoms or signs	Presence of documented symptoms/signs e.g. chills, cough, abdominal pain, headache, mucositis (Yes=1; No=0)	At febrile neutropenia onset	Predictor
Microbiological	Type of organism	Bacterial, fungal, mixed, or not identified	During index febrile neutropenia episode	Descriptive; not available at moment of scoring
Microbiological	Microbiological site of infection	Blood, lungs, perianal, abdomen, central line, urine, or combinations	During index febrile neutropenia episode	Descriptive; not available at moment of scoring
Microbiological	Blood culture positivity	Blood culture positive (Yes/No) and organism code if positive	During index febrile neutropenia episode	Descriptive; not available at moment of scoring (culture results take 1-3 days)
Microbiological	Multiplex PCR positivity	Positive (Yes/No) and organism code, when performed	During index febrile neutropenia episode	Descriptive; not available at moment of scoring
Microbiological	Bronchoalveolar lavage culture positivity	Positive (Yes/No) and organism code, when performed	During index febrile neutropenia episode	Descriptive; not available at moment of scoring

Microbiological	Urine culture positivity	Positive (Yes/No) and organism code	During index febrile neutropenia episode	Descriptive; not available at moment of scoring
Microbiological	Polymicrobial infection	Presence of polymicrobial infection (Yes/No) based on combined culture results	During index febrile neutropenia episode	Descriptive; not available at moment of scoring
Treatment-response	First-line empirical antibiotic regimen	Coded according to predefined antibiotic categories	At febrile neutropenia onset	Descriptive
Treatment-response	Antimicrobial change within 72 hours	Any change or escalation of antimicrobial therapy within 72 hours (Yes/No)	First 72 hours after febrile neutropenia onset	Descriptive; not available at moment of scoring
Treatment-response	Breakthrough fever within 7 days	New fever episode within 7 days after initial febrile neutropenia onset (Yes/No)	First 7 days after febrile neutropenia onset	Descriptive; not available at moment of scoring
Treatment-response	Recurrent fever after 7 days	Recurrent fever after 7 days from febrile neutropenia onset (Yes/No)	Beyond 7 days after febrile neutropenia onset	Descriptive; not available at moment of scoring
Outcome or organ support	Date of sepsis onset	Calendar date on which Sepsis-3 criteria were first fulfilled	During index admission	Outcome
Outcome or organ support	Duration of sepsis	Number of days meeting sepsis criteria	During index admission	Outcome
Outcome or organ support	Intensive care unit transfer	Transfer to ICU (Yes=1; No=0) and coded reason for transfer	During index admission	Outcome

Outcome or organ support	Intensive care unit length of stay	Days spent in ICU	During index admission	Outcome
Outcome or organ support	Ventilatory support	Need for invasive/non-invasive ventilation (Yes/No) and duration	During index admission	Outcome
Outcome or organ support	Renal replacement therapy	Dialysis required (Yes/No) and duration	During index admission	Outcome
Outcome or organ support	Inotropic support	Inotropes required (Yes/No) and duration	During index admission	Outcome
Outcome or organ support	Date of ANC recovery	Date on which ANC exceeded 1500 cells/μL	During index admission	Outcome
Outcome or organ support	Hospital length of stay	Number of days from admission to discharge or death	Index admission	Outcome
