# Supplementary material for: Nurse-Led Sepsis Scoring System for Adult Febrile Neutropenia: Protocol for a Two-Phase Clinical Prediction Model Study With Remote Patient Monitoring
Source: JMIR Res Protoc. 2026 Nov 5;15:e107468. doi: 10.2196/107468 (PMC13740224; doi:10.2196/107468)
Supplement: Supplementary file 2 [file resprot-v15-e107468-s003.pdf]

Multimedia Appendix 3

Outcome adjudication framework and surrogate sepsis criteria

This additional file describes the operational framework that will be used to classify sepsis during the retrospective development phase of the study. The primary outcome is sepsis defined according to Sepsis-3 as suspected or confirmed infection associated with acute organ dysfunction, indicated by an increase of 2 or more points in the Sequential Organ Failure Assessment score from baseline. When pre-illness baseline organ function is unavailable, baseline function will be assumed to be normal unless chronic dysfunction is documented.

Outcome adjudication approach

For each febrile neutropenia episode, sepsis ascertainment will proceed in two stages. First, full retrospective reconstruction of the Sequential Organ Failure Assessment score will be attempted using available clinical, laboratory, and physiological data. If complete reconstruction is not possible because one or more components are unavailable, a predefined surrogate adjudication framework aligned with Sepsis-3 principles will be applied to identify acute infection-associated organ dysfunction.

Surrogate organ dysfunction thresholds

The surrogate thresholds below will be used only when complete reconstruction of the Sequential Organ Failure Assessment score is not feasible. These thresholds are intended to reflect conventional organ dysfunction cut-points that can be identified from routinely available retrospective hospital data.

Organ system	Preferred Sequential Organ Failure Assessment component	Surrogate threshold for retrospective adjudication	Data source
Respiratory	PaO_2/FiO_2 ratio	PaO_2/FiO_2 less than 300, or SpO_2/FiO_2 315 or lower, or new or increased oxygen supplementation or respiratory support	Arterial blood gas, bedside oxygen chart, nursing record
Cardiovascular	Mean arterial pressure or vasopressor use	Systolic blood pressure less than 90 mmHg, mean arterial pressure less than 65 mmHg, or new vasopressor requirement	Vital signs chart, medication record
Renal	Creatinine or urine output	Serum creatinine 2.0 mg/dL or higher, doubling of baseline creatinine where available, clearly documented oliguria, or initiation of dialysis	Laboratory record, intake-output chart

Hepatic	Bilirubin	Total bilirubin 2.0 mg/dL or higher	Laboratory record
Coagulation	Platelet count	Platelet count below 100,000/µL or documented fall of 50% or more from recent baseline	Laboratory record
Central nervous system	Glasgow Coma Scale	New altered mentation or Glasgow Coma Scale score below 15 not attributable to sedation or pre-existing neurological disorder	Medical record, nursing chart

Rules for threshold application

- Surrogate thresholds will be used only when one or more components of the Sequential Organ Failure Assessment score cannot be reliably reconstructed retrospectively.
- Organ dysfunction must be new or acutely worsened during the index febrile neutropenia episode to support outcome adjudication.
- When both full score reconstruction and surrogate criteria are available, the full Sequential Organ Failure Assessment-based definition will take precedence.
- Chronic abnormalities present before febrile neutropenia onset will not be counted as acute organ dysfunction unless acute worsening is documented.

Adjudication algorithm

1. Confirm that the episode meets the study definition of febrile neutropenia based on temperature and neutrophil criteria.
2. Establish the index time of febrile neutropenia onset using the first qualifying fever record and corresponding neutropenia data.
3. Review clinical notes, microbiology records, antibiotic initiation records, and laboratory data to determine whether suspected or confirmed infection was present.
4. Attempt retrospective reconstruction of the Sequential Organ Failure Assessment score using available respiratory, cardiovascular, renal, hepatic, coagulation, and neurological data.
5. If an increase of 2 or more points from baseline is demonstrated, classify the episode as sepsis according to Sepsis-3.
6. If complete reconstruction is not possible, apply the surrogate organ dysfunction thresholds in this additional file to determine whether acute infection-associated organ dysfunction is present.

7. Exclude episodes in which febrile neutropenia and sepsis or septic shock were simultaneously documented at the first febrile neutropenia assessment, because the model is intended to predict progression from febrile neutropenia to sepsis rather than prevalent sepsis at presentation.
8. Record the final outcome classification as sepsis or non-sepsis on the structured adjudication form.

Independent review process

Each retrospective febrile neutropenia episode will be reviewed independently by two adjudicators using the same structured data abstraction sheet and predefined adjudication algorithm. Both adjudicators will be blinded to predictor data where feasible when determining the outcome classification; because predictor values and Sequential Organ Failure Assessment components are both recorded within the same retrospective clinical chart, complete blinding to all predictor information cannot be guaranteed for every episode. Disagreements will be resolved by a third adjudicator or senior clinician, and inter-rater agreement for the binary sepsis classification will be assessed using Cohen's kappa coefficient.

Structured abstraction items

The structured abstraction sheet will include the following items:

- Unique study identification number.
- Date and time of febrile neutropenia onset.
- Evidence supporting suspected or confirmed infection.
- Baseline and worst available values for each Sequential Organ Failure Assessment domain during the relevant assessment window.
- Whether full score reconstruction was possible.
- Whether surrogate organ dysfunction criteria were applied.
- Final classification: sepsis or non-sepsis.
- Reviewer 1 decision, Reviewer 2 decision, and final adjudicated decision.
- Comments explaining uncertain cases or reasons for exclusion.

Phase II adjudication process

Unlike Phase I, in which outcome adjudication is performed independently by two blinded-where-feasible adjudicators, Phase II sepsis outcomes are adjudicated prospectively by the principal investigator using the same Sepsis-3-aligned criteria and structured abstraction approach described above. The principal investigator also calculates the nurse-led index score and the four comparator scores (MASCC, CISNE, NEWS2, and qSOFA) for each Phase II participant. Because outcome adjudication and index-test scoring are performed by the same individual, this process does not achieve independent or blinded assessment of the reference standard relative to the index tests, and no inter-rater

reliability statistic is available for Phase II. This design reflects feasibility constraints given available study personnel at the single study site and is acknowledged as a limitation in the main manuscript.
