# Supplementary material for: Nurse-Led Sepsis Scoring System for Adult Febrile Neutropenia: Protocol for a Two-Phase Clinical Prediction Model Study With Remote Patient Monitoring
Source: JMIR Res Protoc. 2026 Nov 5;15:e107468. doi: 10.2196/107468 (PMC13740224; doi:10.2196/107468)
Supplement: Supplementary file 3 [file resprot-v15-e107468-s004.pdf]

INFORMED CONSENT

I, Ms. Aswathy Asokan., Ph.D. Scholar, Amrita College of Nursing, am conducting a study as part of my thesis titled " Development and validation of a nurse led scoring system for early detection of sepsis among adult patients with febrile neutropenia in a selected hospital from South India." under the guidance of Lt. Col. Prof (Dr) Ajee K L, Professor, Amrita College of Nursing, Kochi. With your permission, I would like to go through your medical records and the medical management you have received. Your participation in the study is voluntary. Your decision to participate will not affect the standard of care. Your request to withdraw from the study at any time and refusal to participate will not involve any penalty or loss.

Being an observational study, your active participation is not required. The method of data collection is observation. You need not undergo any additional procedure or receive any additional drug for the purpose of the study. There is no risk in participation in the study. However, the results of the analysis of the study will help to bring up new findings which may be helpful in the treatment of the same kind of patients in the future.

The information collected will be kept confidential. Your identity will not be revealed in the reports and publications. If you have any questions about the study, you may contact the following person;

Name and contact details of primary investigator:

Ph.D Scholar

Amrita college of Nursing

Contact Mobile Number: 9590047671

Research Guide:

Lt. Col. Prof (Dr) Ajee K L

Professor

Amrita College of Nursing, Kochi.

I ....(Name of the person) undergoing treatment in …………………………with MRD No............................hereby affirm and state as follows.

I have read the written information about the study and confirm that I have had the opportunity to ask questions about this study and I am satisfied with the answers and explanations that have been provided. I understand that my participation is voluntary and that I am free to withdraw at any time, without giving reason. I hereby grant access to data to the investigator described in the information sheet. I voluntarily agree to my participation in the study. I confirm that Ms, Aswathy Asokan has informed me that my data and reports will be used for presentation/scientific publication and research analysis and that such use is for the advancement of medical knowledge. I also understand that my identity will not be revealed in such presentations. I have not given up any of my rights by signing this form.

Signature of the participant: Signature of Father/Mother/Local Guardian:

Address: Address:

Date

Occupation:

Profession:

Signature of witness: Signature of witness:

Name of witness: Name of witness:

Address: Address:

Date: Date:

I have explained and made Mr./Mrs/Ms....................... understand the above - mentioned details of the study.

Signature:

Name:

Address:

Date:
