# Supplementary material for: Nurse-Led Sepsis Scoring System for Adult Febrile Neutropenia: Protocol for a Two-Phase Clinical Prediction Model Study With Remote Patient Monitoring
Source: JMIR Res Protoc. 2026 Nov 5;15:e107468. doi: 10.2196/107468 (PMC13740224; doi:10.2196/107468)
Supplement: Supplementary file 4 [file resprot-v15-e107468-s001.docx]

# Multimedia Appendix 1

## Scoring Rules and Standard Operating Procedure for Nurse-Led and Comparator Risk Scores

This additional file describes the scoring rules and the standard operating procedure that will be used in Phase 2 to calculate the nurse-led index score and the comparator scores: the Multinational Association for Supportive Care in Cancer score, the Clinical Index of Stable Febrile Neutropenia, the National Early Warning Score 2, and the quick Sequential Organ Failure Assessment score. All scores will be calculated using pre-specified rules derived from published definitions, without post hoc modification. Part A presents the scoring rules and item definitions. Part B presents the standard operating procedure governing bedside score calculation in Phase II, including the distinction between the pre-designated primary rater and additional raters participating in the nested inter-rater reliability sub-study.

# Part A: Scoring Rules

## Nurse-Led Index Score

The nurse-led index score is an additive bedside score based on variables routinely available at the onset of the first febrile neutropenia episode. Each predictor is assigned an integer weight, and the total score is obtained by summing the points for all present risk factors, with higher scores indicating greater risk of Sepsis-3 sepsis.

Table 1 presents the provisional variables and scoring thresholds currently specified in this additional file. The final item weights and cut-off values will be confirmed after completion of the re-derived Phase 1 model development under the revised 14-day outcome window, when the final regression coefficients are converted into integer scores using the pre-planned rescaling approach described in the protocol. These weights are therefore provisional pending completion of the re-derived Phase I analysis.

| **Domain** | **Variable** | **Category or threshold used for scoring** | **Provisional points** |
| --- | --- | --- | --- |
| Demographic | Age | Age 60 years or older | 1 |
| Comorbidities | Diabetes mellitus, hypertension, hypothyroidism | Two or more major comorbidities | 1 |
| Vital signs | Heart rate | Greater than 100 beats/minute | 1 |
| Vital signs | Respiratory rate | Greater than 24 breaths/minute | 1 |
| Vital signs | Systolic blood pressure | Less than 100 | 1 |
| Vital signs | Diastolic blood pressure | Less than 80 | 1 |
| Vital signs | Oxygen saturation | Less than 95 | 1 |
| Laboratory values | Absolute neutrophil count | Less than 100 cells/µL | 1 |
| Laboratory values | White blood cell count | Less than 2000 cells/µL | 1 |
| Laboratory values | Procalcitonin | Greater than 0.5 | 1 |
| Clinical signs and symptoms | Mucositis | Present | 1 |
| Clinical signs and symptoms | Abdominal pain | Present | 1 |
| Clinical signs and symptoms | Chills | Present | 1 |

The total nurse-led score will subsequently be used to classify patients into risk categories selected using receiver operating characteristic analysis and clinical judgement.

## Multinational Association for Supportive Care in Cancer (MASCC) Score

The MASCC score will be calculated using the standard published item structure shown below.

| **Variable** | **Scoring rule** |
| --- | --- |
| Burden of illness | Severe = 0; moderate = 3; none or mild = 5 |
| Hypotension | Systolic blood pressure less than 90 mmHg = 0; no hypotension = 5 |
| Active chronic obstructive pulmonary disease | Yes = 0; no = 4 |
| Cancer history | Solid tumour or haematological malignancy without prior fungal infection = 4; otherwise = 0 |
| Dehydration requiring intravenous therapy | Yes = 0; no = 3 |
| Status at onset of fever | Inpatient = 0; outpatient = 3 |
| Age | 60 years or older = 0; younger than 60 years = 2 |

Interpretation: a total score of 21 or higher indicates low risk, whereas a score below 21 indicates high risk.

## Clinical Index of Stable Febrile Neutropenia (CISNE)

The CISNE score will be calculated using the standard published scoring system.

| **Parameter** | **Scoring rule** |
| --- | --- |
| Eastern Cooperative Oncology Group performance status | 2 or higher = 2 |
| Stress-induced hyperglycaemia | Yes = 2 |
| Chronic obstructive pulmonary disease | Yes = 1 |
| Cardiovascular disease history | Yes = 1 |
| National Cancer Institute mucositis grade 2 or higher | Yes = 1 |
| Monocyte count | Less than 200/µL = 1 |

Interpretation: total scores of 0 indicate low risk, 1-2 indicate intermediate risk, and 3 or higher indicate high risk of complications.

## National Early Warning Score 2 (NEWS-2)

The NEWS-2 score will be calculated from respiratory rate, oxygen saturation, supplemental oxygen use, temperature, systolic blood pressure, pulse rate, and level of consciousness using the standard published scoring ranges reproduced below. Total scores will be interpreted according to usual risk categories: 0-4 as low risk, a score of 3 in any single parameter as low-medium risk, 5-6 as medium risk, and 7 or more as high risk.

| **Variable** | **Range** | **Points** |
| --- | --- | --- |
| Respiratory rate, breaths/min | ≤8 | 3 |
| Respiratory rate, breaths/min | 9-11 | 1 |
| Respiratory rate, breaths/min | 12-20 | 0 |
| Respiratory rate, breaths/min | 21-24 | 2 |
| Respiratory rate, breaths/min | ≥25 | 3 |
| SpO2 (room air/supplemental) | ≤91% | 3 |
| SpO2 (room air/supplemental) | 92-93% | 2 |
| SpO2 (room air/supplemental) | 94-95% | 1 |
| SpO2 (room air/supplemental) | ≥96% | 0 |
| Oxygen use | Supplemental oxygen | 2 |
| Oxygen use | Room air | 0 |
| Temperature | ≤35.0°C | 3 |
| Temperature | 35.1-36.0°C | 1 |
| Temperature | 36.1-38.0°C | 0 |
| Temperature | 38.1-39.0°C | 1 |
| Temperature | ≥39.1°C | 2 |
| Systolic BP, mmHg | ≤90 | 3 |
| Systolic BP, mmHg | 91-100 | 2 |
| Systolic BP, mmHg | 101-110 | 1 |
| Systolic BP, mmHg | 111-219 | 0 |
| Systolic BP, mmHg | ≥220 | 3 |
| Pulse, beats/min | ≤40 | 3 |
| Pulse, beats/min | 41-50 | 1 |
| Pulse, beats/min | 51-90 | 0 |
| Pulse, beats/min | 91-110 | 1 |
| Pulse, beats/min | 111-130 | 2 |
| Pulse, beats/min | ≥131 | 3 |
| Consciousness | Alert | 0 |
| Consciousness | New confusion / responds to voice or pain / unresponsive | 3 |

## Quick Sequential Organ Failure Assessment (qSOFA) Score

The qSOFA score will be calculated using three binary variables: altered mental status, respiratory rate of 22 or more breaths per minute, and systolic blood pressure of 100 mmHg or lower. Each positive criterion scores 1 point, giving a total score from 0 to 3. Scores of 0-1 indicate not high risk for in-hospital mortality, whereas scores of 2-3 indicate high risk.

| **Parameter** | **0 Points** | **1 Point** |
| --- | --- | --- |
| Altered mental status, GCS < 15 | No | Yes |
| Respiratory Rate ≥ 22 | No | Yes |
| Systolic Blood Pressure ≤ 100 | No | Yes |

# Part B: Standard Operating Procedure for Bedside Score Calculation (Phase II)

This standard operating procedure governs the bedside calculation of the nurse-led index score in Phase II. It applies to the pre-designated primary rater responsible for each patient's index score, and to additional trained ward nurses participating in the nested inter-rater reliability and bedside feasibility sub-study. Version 2.0, dated on September 2026

## 1. Purpose and Scope

This procedure specifies who calculates the nurse-led score, when it is calculated, from which data sources, how ambiguous or missing values are handled, and how completed scores are recorded and locked. For each eligible patient, the pre-designated primary rater — the staff nurse formally assigned to that patient's care at febrile neutropenia onset — calculates the score used in the primary Phase II diagnostic accuracy analysis. Where at least one additional trained nurse is on duty and available, that nurse (and a third, where rostering permits) independently calculates the nurse-led score for the same patient solely for the nested inter-rater reliability sub-study. These additional scores do not contribute to the primary accuracy analysis. This procedure applies identically to the primary rater and to additional sub-study raters unless otherwise stated. Its purpose is to ensure that the score is applied consistently by different nurses and at different times, so that observed variation in scores reflects genuine differences between patients rather than differences in procedure. Comparator scores (MASCC, CISNE, NEWS-2, qSOFA) are calculated separately by the principal investigator from recorded variables and are outside the scope of this procedure.

## 2. Personnel and Training

Bedside scoring is performed by three rostered staff nurses of the hematology and BMT unit, selected to span the range of unit experience. For each enrolled patient, one of these three nurses — the nurse formally assigned to that patient's care at febrile neutropenia onset — serves as the pre-designated primary rater; the remaining nurses, where on duty and available, participate as additional raters for the nested sub-study only. Each participating nurse completes a single standardized training session before scoring any study patient, comprising:

- Review of this standard operating procedure and the nurse-led item table.
- Item-by-item walkthrough of each score component, including the operational definition, the data source, and the time window for each variable.
- Independent completion of five written case vignettes, followed by review against the answer key.

Competency threshold. A nurse may participate in study scoring — whether as primary rater or as an additional sub-study rater — only after correctly classifying at least [4] of the 5 vignettes into the correct risk category, with no more than [1] item-level error per vignette. Nurses not meeting the threshold receive repeat training and reassessment with an alternative vignette set before participating. The date of training, vignette results, and competency status are recorded on the training log for each nurse.

## 3. Trigger and Timing

Scoring is triggered by the treating team documenting the onset of a febrile neutropenia episode in an enrolled patient. The following windows apply:

| **Step** | **Time window** | **Responsible** |
| --- | --- | --- |
| Primary rater's score sheet initiated | Within 1 hour of documented febrile neutropenia onset | Pre-designated primary rater |
| Primary rater's score sheet completed and submitted | Within 6 hours of febrile neutropenia onset | Pre-designated primary rater |
| Additional sub-study raters' independent parallel scoring | All participating raters (primary and additional) score within the same 60-minute window | Each participating nurse |
| Sheet locked; no further editing permitted | On submission | Automatic / study coordinator |

If the primary rater's score cannot be completed within 6 hours, the sheet is still submitted with the actual completion time recorded, and the delay reason is noted. Out-of-window scores are retained in the dataset and reported separately; they are not discarded. Because ward rostering will not always place all three nurses on the same shift, the number of raters per patient varies between two and three; the achieved number of multiply-rated patients, together with the number rated by three and by two nurses, is reported rather than fixed in advance.

## 4. Data Sources for Each Item

Each item is scored from a single specified source. Nurses do not substitute alternative sources without recording the substitution.

| **Item domain** | **Primary data source** | **Time window** |
| --- | --- | --- |
| Age | Hospital information system, patient record | At admission |
| Comorbidities | Documented medical history in the admission record | At admission |
| Heart rate, respiratory rate | Contactless RPM system reading at onset (Dozee NX-Pro) | Value nearest to onset, within ±[60] minutes |
| Systolic and diastolic blood pressure | RPM-connected device reading | Value nearest to onset, within ±[60] minutes |
| Oxygen saturation | RPM-connected probe reading | Value nearest to onset, within ±[60] minutes |
| Absolute neutrophil count, white blood cell count | Laboratory report | Most recent value within [24] hours before onset |
| Procalcitonin | Laboratory report | Most recent value within [24] hours before onset |
| Mucositis, abdominal pain, chills | Direct patient assessment at the bedside and nursing notes | At the time of scoring |

Where the RPM value and a manually charted value are both available and differ, the RPM value is used, and the discrepancy is noted on the sheet.

## 5. Handling of Missing, Unavailable, or Ambiguous Values

This section exists because inconsistent handling of missing values is one of the most common sources of disagreement between raters, and because per-item missingness is a reported feasibility outcome.

- If a laboratory value is not available within the specified window, the item is recorded as "not available" rather than scored zero. The score sheet records which items were unavailable.
- A score sheet with one or more items recorded as not available is still submitted and retained. The proportion of sheets with any missing item, and the missing rate for each individual item, are reported as feasibility outcomes.
- Nurses do not estimate, impute, or infer values at the bedside. Imputation, where applicable, is handled at the analysis stage under the protocol statistical analysis plan.
- Where an item requires clinical judgement (for example, presence of mucositis), the operational definition in the nurse-led item table (Part A) governs. If the nurse remains uncertain after applying the definition, the item is recorded as "uncertain" with a brief free-text note, and is treated as not present for the total score.
- This rule applies equally to the primary rater and to additional sub-study raters: no nurse consults colleagues, the principal investigator, or other nurses' score sheets when resolving uncertainty.

## 6. Independence and Blinding Requirements for the Sub-Study

- The primary rater and any additional sub-study raters score the patient independently. Sheets are not shared, compared, or discussed before submission.
- Sheets are sealed on submission. Reconciliation or amendment of a submitted sheet is not permitted under any circumstances.
- All participating raters (primary and additional) score within the same 60-minute window, so that differences between raters reflect interpretation rather than genuine change in the patient's condition.
- Nurses are not informed of the patient's subsequent sepsis outcome before submitting a score sheet.
- Completed score sheets — from the primary rater and from any additional sub-study raters — are stored in an access-restricted file separate from the clinical extract supplied to outcome adjudicators.
- Additional sub-study raters' scores are used solely to assess inter-rater agreement and feasibility; they do not contribute to the primary Phase II diagnostic accuracy analysis, discrimination, calibration, or risk classification under any circumstance.

## 7. Recording, Locking, and Audit Trail

Each score sheet — whether completed by the primary rater or an additional sub-study rater — records: participant study identifier; date and time of febrile neutropenia onset; date and time the sheet was started and submitted; the scoring nurse's study identifier and rater role (primary or additional); the value or category recorded for each item; the item points awarded; the total score; the resulting risk category; any items recorded as not available or uncertain; and the time taken to complete the score in minutes. On submission the record is locked and cannot subsequently be edited. An audit trail of entry and submission times is retained for the duration of the study, and the proportion of primary-rater scores submitted within the 6-hour window is reported.

## 8. Feasibility Data Captured at the Time of Scoring

- Time to complete the score, in minutes, from initiation to submission, recorded for the primary rater and for each additional sub-study rater.
- Number and identity of items recorded as not available or uncertain.
- Whether the primary rater's score was submitted within the 6-hour window, and if not, the recorded reason.
- The achieved number of multiply-rated patients, and the number rated by two versus three nurses, reported as part of the sub-study feasibility outcomes.

## 9. Role Boundaries

Nurses performing bedside scoring — whether as primary rater or additional sub-study rater — take no part in adjudication of the primary sepsis outcome. Outcome adjudicators take no part in bedside scoring. The principal investigator calculates comparator scores and takes no part in outcome adjudication. No individual performs both index-test scoring and reference-standard adjudication for the same participant.

## 10. Deviations and Procedure Amendments

Any deviation from this procedure is recorded on the score sheet or the study deviation log at the time it occurs, with the reason. Deviations are summarized in the final report. Substantive amendments to this procedure during the study period are version-controlled, dated, and subject to the ethics-review provisions stated in the protocol. Where item-level Fleiss' kappa falls below 0.60, the affected item may be reworded or re-operationalized in the final scoring manual; any such change is documented as a protocol-specified refinement, with the date of the change and the number of patients scored under each version reported.
